# Advances of cancer-associated fibroblasts in liver cancer

**DOI:** 10.1186/s40364-022-00406-z

**Published:** 2022-08-16

**Authors:** Hao Peng, Erwei Zhu, Yewei Zhang

**Affiliations:** 1 Medical School, Southeast University, Nanjing, 210009 China; 2 The Second People’s Hospital of Lianyungang (The Oncology Hospital of Lianyungang), Lianyungang, 222006 China; 3 Hepatopancreatobiliary Center, The Second Affiliated Hospital of Nanjing Medical University, Nanjing, 210009 China

**Keywords:** Liver cancer, cancer-associated fibroblasts, HCC, CCA

## Abstract

Liver cancer is one of the most common malignant tumors worldwide, it is ranked sixth in incidence and fourth in mortality. According to the distinct origin of malignant tumor cells, liver cancer is mainly divided into hepatocellular carcinoma (HCC) and cholangiocarcinoma (CCA). Since most cases are diagnosed at an advanced stage, the prognosis of liver cancer is poor. Tumor growth depends on the dynamic interaction of various cellular components in the tumor microenvironment (TME). As the most abundant components of tumor stroma, cancer-associated fibroblasts (CAFs) have been involved in the progression of liver cancer. The interplay between CAFs and tumor cells, immune cells, or vascular endothelial cells in the TME through direct cell-to-cell contact or indirect paracrine interaction, affects the initiation and development of tumors. Additionally, CAFs are not a homogeneous cell population in liver cancer. Recently, single-cell sequencing technology has been used to help better understand the diversity of CAFs in liver cancer. In this review, we mainly update the knowledge of CAFs both in HCC and CCA, including their cell origins, chemoresistance, tumor stemness induction, tumor immune microenvironment formation, and the role of tumor cells on CAFs. Understanding the context-dependent role of different CAFs subsets provides new strategies for precise liver cancer treatment.

## Background

Liver cancer causes 841,000 new cases and 782,000 deaths every year, making it the sixth most commonly diagnosed cancer and the fourth leading cause of cancer death worldwide [1]. Primary liver cancer includes HCC (comprising of 75%-85%) and CCA (comprising of 10%-15%) or a mixed form of HCC and CCA. In developing countries, such as China and Eastern Asia, the main risk factor for HCC is hepatitis B virus (HBV) infection, while in developed countries, it is mainly hepatitis C virus (HCV) and alcoholic cirrhosis [2]. Recently, nonalcoholic fatty liver disease (NAFLD) and nonalcoholic steatohepatitis (NASH) are rising in rank as contributors to HCC development [3]. The presence of these etiologies can lead to and exacerbate the progression of liver cirrhosis, and one-third of patients with cirrhosis will develop HCC during their lifetime [4]. Current treatment strategies for liver cancer include hepatic resection, liver transplantation, and systemic chemotherapy (e.g., sorafenib or Lenvatinib), or a combination of different treatment modalities [5–8]. For example, a recent clinical study showed that the combined application of molecular-targeted agents (bevacizumab) and immune checkpoint inhibitors (atezolizumab) has been shown to improve overall survival rates relative to sorafenib in HCC [9]. However, despite great therapeutic advances, the overall prognosis of HCC remains poor.

Although tumor cells hold the main role in driving carcinogenesis, an increasing interest has been focused on the TME. TME includes the cellular and the non-cellular components in solid tumors. The cellular compartment is composed of surrounding stromal cells, immune cells, and angiogenic endothelial cells. The non-cellular component contains extracellular matrix (ECM), various growth factors, chemokines, and cytokines [10]. CAFs constitute the main components of tumor stroma that are closely associated with tumor initiation, progression, stemness, chemoresistance, and prognosis [11]. CAFs can directly communicate with tumor cells and other stromal cells in a paracrine manner or remodel the ECM structure to create a microenvironment conducive to tumor cell invasion and metastasis, which indirectly leads to tumor progression. However, increasing evidence has demonstrated that CAFs do not always exert a tumor-supportive role in oncogenesis, they may also play a tumor-suppressive effect that is context-dependent, namely phenotypic heterogeneity and functional diversity. For example, Meflin-positive fibroblasts could form a tumor inhibitory CAFs subpopulation in pancreatic ductal adenocarcinoma (PDAC), which are correlated to favorable outcomes [12]. The diversified cell origin of CAFs may partly contribute to the heterogeneity of CAFs function, and the phenotypic switch of CAFs under the corrupting influence of TME during tumor evolution may be another reason for the heterogeneity. Therefore, understanding the dynamic communication between various cells in the tumor, especially how the reciprocal crosstalk between CAFs and other cells reshapes the TME, is essential for comprehending and treating liver cancer from an evolutionary and holistic perspective. In this review, we discuss the heterogeneous cellular origin of CAFs, their functional malleability in HCC and CCA, respectively, and mainly focus on the latest research progress of CAFs interacting with various cellular components in the TME of the liver cancer.

## The characterizations and cellular origin of CAFs in HCC

### The unique characteristics of CAFs

Liver normal stroma cells constitute the connective tissues that supply a supportive framework for the liver tissues. Among the stromal components, normal fibroblasts produce a variety of collagens or matrix metalloproteinase (MMP) to maintain the integrity of ECM or remodel the ECM to keep the matrix components in a dynamic equilibrium [13]. When the liver is stimulated by various stimuli in the context of liver cirrhosis or in the process of tumor development, normal fibroblasts or other types of cells can be activated into myofibroblasts or cancer-associated fibroblasts, exhibiting enhanced secretory function and ECM accumulation.

**Fig. 1:**
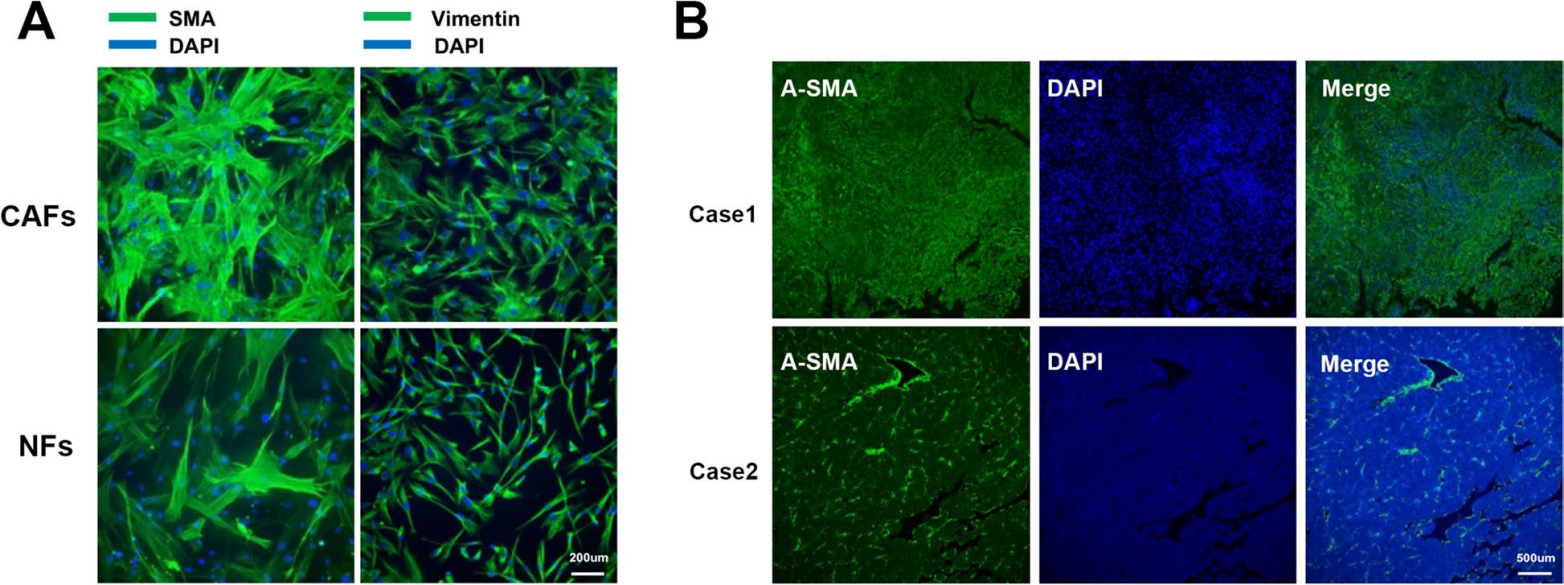
Morphological manifestations of fibroblasts in HCC. **A** CAFs and NFs extracted from HCC tissue and normal liver tissue were identified by immunofluorescence staining for α-SMA and Vimentin. CAFs exhibited more abundant cytoplasmic content than NFs. **B** Representative graphs showed two HCC cases with different α-SMA + CAFs distribution densities, with case1 exhibiting more rich CAFs infiltration relative to case2. Peng, H., R. Xue, Z. Ju, J. Qiu, J. Wang, W. Yan, et al., Ann Transl Med, 2020. 8(14): 856

CAFs are easy to isolate from fresh liver cancer tissues and to culture in vitro over several passages with stable phenotype. Different from the normal fibroblasts (NFs), CAFs are identified by spindle-shaped morphology but with multiple branches of cytoplasm, larger indented nuclei, plentiful ribosomes, increased rough endoplasmic reticulum, and a well-developed Golgi apparatus [14, 15]. α-smooth muscle actin (α-SMA) is expressed by multiple CAFs subsets, that is usually be used to identify CAFs in liver cancer [16]. Other reported proteins such as fibroblast activation protein (FAP), vimentin, platelet-derived growth factor (PDGF) receptor (PDGFR)-α and β, and fibroblast-specific protein 1 (FSP-1) can also serve as markers of CAFs [17]. In our previous study, the extracted CAFs were identified by immunofluorescence staining of α-SMA and Vimentin, and the morphological characteristics of CAFs could be observed [18], as shown in Fig. 1A. The distribution of CAFs in HCC tissues can be aggregated, sporadic, and localized along hepatic sinusoids, Fig. 1B showed two different distributions of CAFs in HCC. Moreover, studies have shown that the abundance of CAFs positively correlated with tumor size, and the higher the density of CAFs, the worse the prognosis of HCC patients [19, 20]. Nevertheless, due to the lack of specific lineage biomarkers, it remains a challenging topic in studying CAFs in vivo.

Functionally, activated CAFs exhibit enhanced secretory phenotype, secreting various cytokines including transforming growth factor-β (TGF-β), hepatocyte growth factor (HGF), insulin-like growth factor (IGF), interleukin-6 (IL-6) et al., which can, in turn, induce the proliferation, migration, immune response of liver cancer cells [21]. Apart from this, one of the hallmarks of CAFs is their high capacity for ECM synthesis, including fibronectin as well as different types of collagens [22]. Unbalanced synthesis and degradation of local ECM induce mechanical stiffening of the tissues, which may modulate tumorigenesis and influence the prognosis of patients. In general, CAFs communicate with various cells in the TME by producing various cytokines and ECM proteins to construct a microenvironment suitable for tumor growth and dissemination.

### The cellular origin of CAFs in HCC

Emerging studies demonstrate that CAFs can be derived from multiple cell types, including normal resident tissue fibroblasts, hepatic stellate cells (HSCs), mesenchymal stem cells (MSCs), epithelial or endothelial cells [23]. The genetic fate-mapping technique using lecithin retinol acyltransferase-cyclization recombination enzyme (Lrat-Cre) and PDGF receptor beta (PDGFRB)-Cre determine HSCs as the dominant source of myofibroblasts in liver fibrosis [24, 25]. HSCs are commonly residing in the perisinusoidal space and occupy 15% of resident cells in the liver, their unique function is to store vitamin A [26]. Under the stimulation of several cytokines, HSCs can undergo a morphophysiological transformation and acquire a myofibroblast-like phenotype, manifested by increased α-SMA expression and enhanced collagen secretion [27]. TGF-β plays an essential role in all stages of liver disease progression, from inflammation to cirrhosis and liver cancer. During tumorigenesis, TGF-β is a well-known growth factor that can activate HSCs into α-SMA ( +) CAFs [28]. Another research revealed that hepatocyte-derived PDGF-C could also transform HSCs into myofibroblast-like cells to accelerate the progression of HCC [29].

The application of the latest genetic tracing and single-cell RNA sequencing (scRNA-seq) approaches provides more favorable evidence to trace the origin of CAFs. In a recent study, the authors analyzed scRNA-seq data from mouse liver cells and found transcription factor 21 (Tcf21) could be identified as a specific marker that distinguished quiescent HSCs from activated HSCs. By tracing Tcf21 positive cells, they observed these cells mainly marked periportal and pericentral HSCs, Tcf21 positive HSCs were quiescent under steady-state but became activated in the DEN/CCL4-induced state, generating 85% of CAFs in liver tumors [30]. Another recent study using genetic tracing in combination with scRNA-seq analysis demonstrated that liver metastasis-associated CAFs are primarily HSCs derived, as the majority of CAFs showed abundant expression of HSCs signature that included HSC markers [31].

MSCs are multipotent cells, which can self-renew and differentiate into adipocytes, cartilage, bone, and other cells under the appropriate conditions [32]. MSCs can be recruited to the stroma of HCC and play an essential role in HCC initiation, progression [33]. MSCs infiltrating the TME can be transformed into CAFs-like cells after being acclimated by surrounding tumor cells. For example, a recent study investigated that when co-cultured with Huh7 cells, MSCs significantly upregulated the expression of CAFs markers (α-SMA, Vimentin, c-MYC, MMP2, VEGF, IL-6, FGFR1, IL-8, Tenascin-C), thereby acquiring the CAFs-like phenotype and characteristics [34]. Another study revealed after exposure to SK-Hep1, MSCs can also exhibit the properties of CAFs [35]. However, both studies are only limited to cell lines co-cultivation assays in vitro, the in vivo lineage-tracing experiments are required to determine the conversion of MSCs to CAFs in the future.

Additionally, epithelial cells through epithelial-mesenchymal transition (EMT) or endothelial cells undergoing endothelial to mesenchymal transition (EndMT) can acquire mesenchymal properties, which might serve as another source of CAFs [36, 37]. For example, Zeisberg et al. have revealed that TGF-β induced adult mouse hepatocytes to undergo phenotypic and functional switch typical of EMT, which contributed to the population of FSP1-positive fibroblasts in CCL_4_-induced liver fibrosis [37]. However, this origin may be controversial, another research utilizing triple transgenic mice demonstrated that hepatocytes in vivo neither acquire mesenchymal marker expression nor exhibit a myofibroblasts-like morphology [38]. In some specific contexts, HCC cells may undergo EMT and express markers of CAFs. For instance, hypoxic conditions can induce upregulation of FAP expression in HCC cells [39]. Another study explored that TGF-β promoted α-SMA expression in HCC cells [40]. A recent study using the in vitro EndMT model found that after treatment with TGF-β1 and IL-1β, human fetal liver sinusoidal endothelial cells tend to transition to fibroblast-like cells, while mesenchymal markers were increased, and endothelial markers were decreased [41]. However, these studies are limited to the expression of surface markers of CAFs and lack the exploration of the functional features of CAFs in vivo.

In addition to the above possible cell sources, portal fibroblasts (PFs) as a small population of “periductular mesenchymal cells” can also be an origin of CAFs [42]. Lysophostatidic acid (LPA) secreted by HCC can act on PFs to convert them into CAFs, after transdifferentiation, PFs acquired the expression of α-SMA and enhanced the proliferation, migration, and invasion of HCC [31, 43]. This source awaits more research to prove in the future.

## The roles of CAFs in HCC progression

Numerous evidence has shown that CAFs are involved in the regulation of tumor cell proliferation, invasion, migration, metastasis, etc. In the treatment of HCC, CAFs promote tumor chemoresistance and recurrence. By secreting various forms of cytokines, growth factors, or extracellular vesicles, CAFs communicate closely with HCC cells either directly or indirectly. Recent data based on spatial proteome profiling of HCC cells and CAFs further supports the interaction between CAFs and HCC cells. For instance, CAFs might release BGN, VCAN, which bind to the receptor TLR2 on the surface of HCC cells in a paracrine way to initiate downstream signal transduction. Alternatively, CD81 expression on CAFs can directly bind to GPC3 on HCC cells, affecting the biological behavior of both [44].

Nevertheless, considering the heterogeneity of CAFs, they might not always play a tumor-promoting role in the TME, on the contrary, they may also exert a tumor-restraining effect in tumor growth. A recent study showed that some specific CAFs subgroups in the TME can exert a mutually antagonistic effect in HCC. By analyzing scRNA-seq data, CAFs in liver metastasis can be divided into three subgroups that are myofibroblastic CAFs (myCAFs), inflammatory CAFs (iCAFs), and PF/mesothelial CAF (PF/mesCAF). myCAFs expressing type I collagen could suppress tumor growth in a manner of mechanical restriction [31]. Another study based on proteomic and scRNA-seq analysis showed that HCC-infiltrating CAFs can be divided into three subtypes [45], the heterogeneous population of CAFs in HCC was summarized in Table 1.

**Table 1:** CAFs subclusters identified in liver cancer

Sample origins	CAFs subclusters	Signature genes or characteristics	Genes enrichment pathway
HCC [45]	CAF_VSMC	RGS5, NDUFA4L2, MYH11,CNN1	signatures of smooth muscle vascular cells
	CAF_HSC	PDGFRB, THY1	signatures of hepatic stellate cells
	CAF_Port	PDGFRA, MMP23B, COL1A1, PRELP	signatures of portal fibroblasts
ICC [46]	myCAF	COL1A1, ACTA2, COL8A1, COL15A1, CRLF1, FBN2, SERPINF1	ECM pathways;
			ICC proliferation;
			intraneural invasion
	iCAF	CCL19 CCL21, IL6, RGS5	inflammatory, growth factor; antigen-presentation genes;
			receptor-ligand, growth factor, and cytokine activity pathways
	mesCAF	ANXA1, ANXA2, CXCL1 CXCL6	mesothelial markers
ICC [47]	Subcluster 0 (vCAFs)	CD146, MYH11, GJA4, RGS5, IL-6, CCL8	microvessels and inflammatory chemokines signature
	Subcluster 1 (mCAFs)	COL5A1, COL5A2, COL6A3, POSTN, FN1, LUM, DCN, and VCAN	ECM pathways;
			collagen fibril organization
	Subcluster 2 (iCAFs)	FBLN1, IGFI, CXCL1, IGFBP6, SLPI, SAA1, C3, C7	inflammatory response regulation;
			complement activation
	Subcluster 3 (apCAFs)	CD74, HLA-DRA, HLA-DRB1	leukocyte cell–cell adhesion; response to IFN-c;
			antigen processing;
			antigen presentation via MHC-II
	Subcluster 4 (eCAFs)	KRT19, KRT8, SAA1	EMT
	Subcluster 5 (lipofibroblast)	APOA2, FABP1, FABP4, and FRZB	lipid metabolism and processing
Liver metastasis [31]	myCAF	ACTA2, COL1A1, COL1A2, COL1A3, COL15A1, MMP2	ECM pathways
	iCAF	HGF, BMP10, GDF2, LFITM1	growth factor and inflammatory genes
	mesCAF	MSLN, UPK1B, UPK3B, GPM6A	mesothelial markers

### CAFs enhance chemoresistance of HCC cells

Resistance to anti-tumor therapeutics often leads to tumor progression. Sorafenib and Lenvatinib have been administrated for patients with advanced HCC, however, the median overall survival (OS) with either sorafenib or Lenvatinib is only about 13 months [7, 8]. The interaction between CAFs and HCC is one of the potential mechanisms that weaken the sensitivity of tumor cells to chemotherapeutic drugs. Our previous research showed CAFs secreted HGF to upregulate the expression of cell differentiation (CD)-73, and CD73 positive HCC cells were more resistant to the effects of sorafenib and cisplatin [18]. Additionally, a recent study by our team showed that CAFs can induce the Reticulocalbin 1 (RCN1) expression in HCC, and high-expressing RCN1 can attenuate the sensitivity of HCC cells to sorafenib via the IRE1α-XBP1s pathway [48]. Apart from that, chemoresistant HCC cells can also promote CAFs functional enhancement, which in turn provides a more favorable microenvironment for HCC cells to survive. For example, when co-cultured with sorafenib-resistant HCC cells, CAFs can be activated through the B-cell activating factor (BAFF) / NF-κB pathway to further enhance the chemoresistance of HCC [49].

### The influence of CAFs on the stemness of HCC cells

Cancer stem cells (CSCs) are categorized by their enhanced self-renewal properties and multilineage differentiation, this population of cells confer resistance to therapy and facilitate the metastasis, recurrence of HCC [50]. In HCC progression, CAFs can provide a favorable tumor niche to support CSCs survival and sustain their stemness in a paracrine manner.

Prior studies have elucidated that HGF, as a mediator in the conditioned media of CAFs, paly a major role in the induction of liver CSCs. Mechanistically, CAFs-derived HGF enhanced stemness through the extracellular signal-regulated kinase (ERK)1/2–FRA1–HEY1 signaling pathways in HCC [51]. In addition to its direct role on stemness maintenance, HGF can also further enhance the stemness capacity of HCC by upregulating the expression of stemness-related molecules. CD73 positive HCC cells have been demonstrated to exert stemness maintaining function by upregulating SOX9 expression and preventing its protein degradation [52]. Our previous study revealed that CAFs could secret the amount of HGF to enhance the sphere-forming capacity of CD73 positive HCC cells via the MET-ERK1/2 pathway [18]. Consistently, Keratin19 has been determined to be a CSCs marker in HCC [53], and it can be induced by HGF from CAFs via a MET-ERK1/2-AP1 and SP1 axis [54]. Another study indicated that CAFs-derived IL-6 promoted stem cell-like properties in HCC cells by enhancing STAT3/Notch signaling [55]. The cluster of differentiation24 (CD24) has previously been identified as another CSCs marker in liver cancer [56]. CD24 positive HCC cells possessed a high capacity of self-renewal, and CAFs-secreted HGF and IL6 enhanced stemness of CD24 positive HCC cells through activated STAT3 pathway [57]. Peri-tumor fibroblasts also produced more IL-6, which induce HCC stemness via IL-6-STAT3-pathway [58]. Moreover, peri-tumor tissue-derived fibroblasts could also recruit CSCs to maintain their stemness via generating a series of cytokines [59].

Besides, other cytokines have recently been reported to regulate HCC stemness. Follistatin-like 1 (FSTL1), a pro-inflammatory factor, which is found to be upregulated during inflammation, is predominantly secreted from the CAFs rather than liver cancer cells. FSTL1 binding to the TLR4 receptor on HCC cells could augment the stemness through deregulated AKT/mTOR/4EBP1 signaling pathways [60]. Additionally, cardiotrophin-like cytokine factor 1 (CLCF1), a cytokine that belongs to the IL-6 superfamily, can be secreted by CAFs to induce the secretion of (C-X-C motif) ligand 6 (CXCL6) and TGF-β in HCC, which subsequently promote tumor cell stemness in an autocrine manner. Furthermore, CXCL6 and TGF-β could activate the CAFs through ERK1/2 signaling to generate more CLCF1 to further sustain the stemness of HCC [61]. However, the liver X receptors (LXRs) can inhibit the expression of key markers in CAFs, thus limiting the differentiation of CAFs. Activation of LXRs can antagonize the effect of TGFβ1-induced CAFs on hepatosphere formation [40].

Previous studies have revealed that FOX members can enhance stem cell-like characteristics in cancers [62]. Luo et al. demonstrated that FOXQ1 was induced by CAFs in co-culture systems in vitro and in vivo, and HCC initiation was promoted by CAFs via FOXQ1/NDRG1 axis. Moreover, the activated FOXQ1/NDRG1 axis could feedback to the recruitment of infiltrating CAFs through the pSTAT6/CCL26 signaling pathway, thus, further enhancing the initiation of HCC [63]. Increasing evidence shows that autophagy is essential in the maintaining of stemness in liver CSCs [64, 65]. Zhao et al. found that the potential mechanism of CAFs to promote HCC stemness may be mediated by autophagy via the mTOR pathway in HCC [66]. The Notch signaling pathway also plays an important role in promoting the self-renewal of liver CSCs [67]. CAFs could maintain CSCs stemness by activating Notch3/LSD1 signaling [68]. Resolvin D1 (RvD1) is an endogenous anti-inflammatory lipid mediator, which targets stromal cells to exert an anti-tumor effect. RvD1 could suppress the cartilage oligomeric matrix protein (COMP) secreted by CAFs, thus, abrogating the promoting effects of CAFs on stemness in HCC via FPR2/ROS/FOXM1 signaling [69].

### CAFs shape tumor immune microenvironment in HCC

Tumor-infiltrating lymphocytes (TILs) are a highly heterogeneous population of immune cells that exert pivotal roles in immune evasion and response to immunotherapy [70]. Accumulating previous studies have confirmed the critical roles of the crosstalk between CAFs and TILs in tumorigenesis and progression. In HCC, TILs include innate immune cells and adaptive immune cells, the former contains tumor-associated macrophages (TAMs), tumor-associated neutrophils (TANs), NK cells, and DC cells, while the latter is composed of T lymphocytes, CD4 + /CD8 + T cells, regulatory T cells (Tregs), and myeloid-derived suppressor cells (MDSCs) [71]. The tumor immune microenvironment (TIME) constituted by these immune cells determines the state of the immune response, and numerous studies have reported that CAFs promote tumor immune escape by influencing the proportion and activity of TIME.

TAMs are abundant infiltration in HCC, these cells can be divided into two subpopulations: M1 macrophages and M2 macrophages. M1 macrophages can be polarized by lipopolysaccharides (LPS), interferon-γ (IFN-γ), or tumor necrosis factor (TNF), to exert pro-inflammatory function, while IL-4, IL-10, and IL-13 lead to the M2 polarization with immunosuppressive function [72]. The research on the function of M1-polarized macrophages is limited in HCC. In the TME of HCC, CAFs tend to promote the M2 polarization of macrophages, possibly mediated by the secretion of IL6 [73]. Moreover, CAFs could generate CXCL12 to induce the secretion of plasminogen activator inhibitor‑1 (PAI‑1) in TAMs, and the up-regulation of PAI-1 in TAMs accelerated the malignant progression of HCC [74]. Endosialin is a member of the C-type lectin-like receptor family and is specifically expressed in cancer cells and tumor stromal cells [75, 76]. Recently, a study demonstrated that Endosialin was mainly expressed in CAFs, endosialin-positive CAFs could recruit the TAMs through interaction with CD68 in TAMs and secrete growth arrest-specific protein 6 (GAS6) to mediate the M2 polarization to promote the HCC progression [77].

Similar to TAMs, TANs also exhibit a dual role in HCC, they can be either anti-tumorigenic (N1) or pro-tumorigenic (N2) [78]. In HCC, CAFs could induce chemotaxis of neutrophils through the stromal cell-derived factor (SDF)-1a/CXCR4 pathway and promote PDL1 expression in neutrophils, the recruited neutrophils exerted immunosuppressive function by inhibiting the T cell immunity via the IL6-STAT3-PDL1 signaling pathway [79]. As mentioned above, in addition to sustain the stemness of HCC, CLCF1 produced by CAFs could also enhance TANs infiltration and polarization through increasing the secretion of CXCL6 and TGF-β in HCC cells [61].

NK cells as members of the innate immune system, which initiate anti-tumoral cytotoxic in various solid tumors. HCC patients often displayed reduced numbers of NK cells in the peripheral compared with healthy subjects and exhibited poor capacity to kill tumor cells [80]. CAFs tend to inhibit the activation and cytotoxic activity of NK cells in the HCC microenvironment. For example, CAFs secreted prostaglandin E2 (PGE2) and indoleamine 2,3-dioxygenase (IDO) could deactivate the NK cells and attenuate their cytotoxic activity, thereby forming an unresponsive niche for HCC progression [81].

Tumor-infiltrating DCs are essential in the activation of naïve T cells and initiate the adaptive immune response against tumors in the TIME. Moreover, there exists a population of regulatory DCs (rDC), which promote T cell anergy and induce Treg differentiation, thus contributing to immunotolerance in HCC [82]. CAFs are capable of attracting normal DCs into the tumor site through secreting SDF-1α and educating them to acquire tolerogenic characteristics, which resemble rDC. These CAFs-educated rDC cells highly expressed IDO, showing strong immunosuppression of T cell response and facilitating the proliferation of CD4 + CD25 + Foxp3 + Treg cells via IL-6-mediated STAT3 activation [83]. However, whether CAFs can directly recruit and activate infiltrating Treg cells has not been studied, which deserves further investigation in the future.

MDSCs are a highly heterogeneous population composed of two cell subsets: monocytic MDSCs (M-MDSCs) and polymorphonuclear MDSCs (PMN-MDSCs), which are similar to monocytes and neutrophils in phenotypes and morphologies, respectively [84]. MDSCs possess potent immunosuppressive effects. PMN-MDSCs are mainly enriched in TIME and generate large amounts of reactive oxygen species (ROS) to mediate T cell tolerance. However, M-MDSCs accumulate in peripheral blood expressing high levels of iNOS but releasing low ROS to suppress the immune response [85]. Emerging studies have shown that CAFs could promote the generation of MDSCs through paracrine manner. In HCC, CAFs recruit monocytes into TIME by SDF-1a/CXCR4 pathway, then induce the monocytes to differentiate into CD14 + HLA-DR-/low MDSCs, which depended on IL-6/STAT3 manner. These educated MDSCs could impair T cells function to block the anti-tumor immune response [86]. Another research also confirmed that CAFs could produce higher levels of chemokines and cytokines, such as macrophage colony stimulating factor (M-CSF), monocyte chemotactic protein-1(MCP-1), and TGFβ1, to recruit the MDSCs into the TIME, however, the specific molecular mechanism needs to be further studied [87]. Additionally, activated HSCs could also promote the accumulation of MDSCs in HCC by releasing cyclooxygenase-2 (COX-2) and PGE2 [88]. The interaction between CAFs and TIL in liver cancer was summarized in Table 2

**Table 2:** Interactions between CAFs and TILs in the TME of liver cancer

	**Immune cells**	**CAFs-secreted factors**	**Mechanisms**	**Phenotype**	**Reference**
**HCC-CAFs**	TAMs	CXCL12;	CXCL12/CXCR4–PAI-1;	M2 polarization of TAMs;	[74];
		GAS6	Endosialin-CD68	macrophage recruitment and polarization	[77]
	TANs	SDF1a;	IL6/STAT3-PDL1;	Chemotaxis of TAN;	[79];
		CLCF1	CLCF1 − CXCL6/TGF-β	TAN infiltration and polarization	[61]
	NK cells	PGE2, IDO	-	Inducing deactivation of NK cells	[81]
	DCs	SDF-1α	IL-6/STAT3-IDO	Induction into rDC / promotion of Tregs expansion	[83]
	MDSCs	SDF1a;	IL-6/STAT3;	Inducing monocytes to differentiate into MDSCs / Impairing T cells function	[86];
		M-CSF, MCP-1;	-;		[87];
		COX-2; PGE2	ERK/COX2/PGE2		[88]
**CCA-CAFs**	MDSCs	CCL2;	FAP-STAT3-CCL2;	Recruitment of MDSC;	[89];
		IL-6, IL-33	5-LO/LTB4-BLT2	Enhancing the stemness capacity of MDSCs	[90]
	TANs	CXCL5	PI3K-AKT and ERK1/2	Recruitment of CD66b + TANs	[91, 92]
	Tregs	-	-	CAV1 + CAFs positively correlated with Foxp3 + TIL	[93]

### CAFs facilitate HCC angiogenesis

HCC is a hypervascularized tumor, and neo-angiogenesis leads to tumor cells dissemination, invasion, disease recurrence, and metastasis. Besides, tumor angiogenesis is a dynamic process that is mediated by pro-angiogenesis factors secretion by the tumor cells and stromal cells in the TME. For instance, CAFs secreted vascular endothelial growth factor (VEGF) in the surrounding tumor sites to promote the proliferation and angiogenesis of human umbilical vein endothelial cells (HUVECs) via enhancer of zeste homolog-2 (EZH2) /vasohibin 1 (VASH1) pathway [94]. In addition to VEGF, the placental growth factor (PlGF) is another angiogenic factor that can promote angiogenesis in HCC [95]. A recent study has shown that CD90 positive CAFs have a strong correlation with PIGF expression in HCC tissues, and highly expressed of CD90 and PIGF in CAFs were related to angiogenesis-related markers in vascular endothelial cells, such as CD31, CD34, and CD105, thus to facilitate the angiogenesis of HCC [96].

Tumor vasculogenic mimicry (VM) is an alternative way that tumor cells establish the blood supply in the absence of HUVECs. In the mouse xenografts model, implantation of CAFs with tumor cells could significantly enhance the VM formation in HCC tissues when compared with implanting tumor cells alone. Mechanistically, CAFs-derived TGF-β and SDF1 facilitated VM formation by inducing the expression of endothelial cells markers and the ECM remodeling-associated genes, such as VE-cadherin, MMP2, and laminin5γ2 in tumor cells [97].

However, in addition to the positive effect on angiogenesis, specific CAFs subsets can also play a opposite role in tumor blood vessels. According to a recent study, prolargin was solely expressed and secreted by a subset of CAFs deriving from portal fibroblasts, which bound and antagonized several pro-angiogenic growth factors, such as FGF1, FGF2, HGF, and TGF-β1, to inhibit the angiogenesis of HCC and was positively correlated with good clinical outcome in HCC patients [45]. SPARCL1 is a secreted protein and plays a tumor suppressor role in several tumors [98, 99]. Another recent scRNA-seq-based analysis found that SPARCL1-positive fibroblasts, which were located in the large blood vessels in the stromal niche of liver tumor, representing a group of vessels associated fibroblasts, could maintain the self-stabilization of blood vessels, thereby reducing tumor cell invasion and is associated with a favorable prognosis for the HCC patients [41]. In general, the above studies reveal that the effect of CAFs on tumor blood vessels is partly attributed to their cell origin or spatial localization. However, little is known about the function of tumor suppressive CAFs, which requires more research on the in vivo spatial transcriptome in the future.

### Activation of CAFs in HCC

A large number of documents have shown that there exists a bi-directional communication between tumor cells and stromal cells, that is, CAFs can not only influence the initiation and progression of the tumor, but tumor cells or other cells in the TME can also stimulate the activation of CAFs, thereby forming the feedback loop further accelerates the deterioration of the tumor.

HCC cells can act on the precursor cells of CAFs, such as HSCs or other progenitors, to activate them in the manner of paracrine or exosomes. TGFβ is a well-researched inflammatory factor that can educate HSCs into myofibroblast-like cells, as mentioned above, HCC-derived TGFβ and CXCL6 could activate CAFs to enhance their secretory function [61]. TGFβ can also stimulate HCC cells to produce more connective tissue growth factor (CTGF), and TGFβ-dependent CTGF secretion can drive tumorigenesis with high stroma infiltration [100]. Mechanistically, CTGF as a matricellular protein related to fibrosis can be secreted by HCC cells to induce adjacent HSCs activation in the TME and this tumor-promoting effect of HSCs can be abolished by anti-CLGF neutralizing antibody [101]. Consistently, hepatocyte-derived PDGF-C promoted the conversion of HSCs into myofibroblasts-like cells by binding to the PDGF receptor located on HSCs [29]. Tissue inhibitor of matrix metalloproteinases (TIMPs) inhibits MMP proteolytic activity and mediates the remodeling of ECM. A study has shown that TIMPs expression in HCC can induce the liver fibroblasts into CAFs and then protect HCC cells from apoptosis via SDF-1/CXCR4/PI3K/AKT signaling pathways [102]. Extracellular sulfatase 2 (SULF2) is a member of sulfatase family genes, when co-cultured with HSCs, SULF2 could be secreted by the HCC cells to induce the differentiation of HSCs into CAFs via the TGFβ1/SMAD3 signaling pathway [103]. Another study has reported upon co-cultured with HCC cells, HSCs produced more HGF and stimulated STMN1 expression via MET pathway in HCC cells, subsequently, STMN1 enhanced PDGF homodimeric protein expression, which might facilitate HSCs activation to acquire CAFs phenotype [104]. More recently, research showed that endoplasmic reticulum (ER) stress also mediated the mutual communication between HSCs and HCC. In the context of HCC, unfolded proteins accumulated and activated ER stress. Inositol requiring enzyme 1α (IREα), as a three-transmembrane protein, can sense the presence of misfolded proteins under ER stress [105, 106]. HCC cells activated IREα in HSCs, thereby leading to HSCs activation in vitro 2D and 3D co-culture systems [107]. Periostin is a matricellular protein involved in collagen deposition, which contributes to the development of various tumors [108]. Periostin can stimulate HSCs activation in an autocrine integrin-FAK-STAT3-periostin circuit and enhance HCC cells proliferation via the ERK pathway in a paracrine manner [109]. Additionally, the sox 9/inhibin subunit beta B (INHBB) axis also plays a critical role between HSCs and HCC cells. Sox9 positive HCC cells induce INHBB expression and activin B secretion, and accordingly promote the activation of surrounding HSCs, ultimately favoring HCC metastasis [110]. In addition to HSCs, PFs can also be activated and converted into myofibroblasts-like phenotypes under the stimulation of LPA secreted by HCC cells [43].

Emerging evidence indicates that CAFs exhibit a senescence-associated secretory phenotype (SASP), that is able to secrete more pro-inflammatory cytokines in HCC. Bone morphogenetic protein 4 (BMP4) is a member of the TGF-β superfamily and is involved in organogenesis in the liver [111]. A study has found that BMP4 was highly expressed in CAFs compared to NFs and produced several SASP factors to enhance tumor invasiveness. Moreover, BMP4 expressed by HCC cells could be acted as an exogenous stimulating factor to exacerbate the activation of CAFs in the HCC microenvironment [112]. Similarly, deoxycholic acid (DCA) can cause the senescence of HSCs and induce the production of SASP factors. Furthermore, the surgical specimens of HCC patients also confirmed that the senescent HSCs were located in the stroma surrounding HCC, revealing the role of bile acid in HSCs activation in TME [113]. Gluconeogenic enzyme fructose 1,6-bisphosphatase 1 (FBP1) as a metabolic tumor suppressor in HCC, can trigger HSCs activation and senescence by releasing HMGB1 after being deleted in hepatocytes, showing a SASP [114].

Exosomes are a subtype of extracellular vesicles, which contain various biological substances that can be secreted from one cell and transferred to another, acting as a carrier for signal transmission [115]. Tumor cells can also promote the activation of HSCs in the form of exosomes [116]. A prior study has shown that exosomal miRNA-21 secreted by HCC cells promoted HSCs activation via PDK1/AKT signaling pathway [117], meanwhile, miRNA-21 could induce the progression of liver cirrhosis to liver cancer by promoting HSCs activation and collagen deposition via the TGF-β signaling pathway [118]. Exosomal miR-1247-3p secreted by high-metastatic HCC cells regulated fibroblasts activation via B4GALT3-β1-integrin-NF-κB axis in lung pre-metastatic niche from liver cancer, and the activated CAFs enhanced the secretion of pro-inflammatory cytokines, thereby promoting the stemness, EMT, chemoresistance, and tumorigenicity of HCC cells [119].

In addition to the above-mentioned stimulatory cytokines, mechanical factors such as matrix stiffness are also involved in CAFs activation. Matrix stiffness can promote malignancies by shaping the biological characteristics of tumor cells via directly regulating their growth and motility [120]. A recent study showed that stiffness induced HSCs activation through the CD36-AKT-E2F3 mechano-signaling pathway, which in turn promoted FGF2 transcription and secretion to promote HCC growth and distant metastasis [121]. Another research also provided the evidence that stiffness promoted HSCs activation relied on p300 nuclear accumulation, which was mechanistically mediated by RHOA-AKT pathway [122]. It is worth noting that both TGF-β-mediated and stiffness-mediated HSCs activation require p300, however, the transcription targets of the two are different [123].

## The characterizations and cellular origin of CAFs in CCA

### The unique features of CAFs in CCA

CCA is the second most frequent primary malignancy of the biliary system in liver cancer [124]. According to the anatomical location, CCA can be subdivided into three distinct subtypes: intrahepatic CCA (ICC), perihilar CCA (PCC), and distal CCA (DCC) [125]. Although CCA is a relatively rare malignant tumor; however, its incidence has increased in the last decade due to a rise in ICC [125]. The three subtypes have different clinical characteristics, therapeutic strategies, and prognosis. Considering the features of intratumor heterogeneity, one subtype can also be divided into different subgroups. A recent scRNA-seq analysis-based study revealed the intratumoral diversity of ICC cells. The malignant cells can be classified by four subclusters: subcluster 0 malignant cells highly expressed markers related to the EMT process, subcluster 1 showed enrichment in cell-cycle and hypoxia-dominant signature, subcluster 2 exhibited high expression of immune-related genes and subcluster 3 malignant cells highly expressed SPINK1, which was closely associated with poor prognosis in ICC [47]. Unlike the histological features of HCC, the most prominent hallmark of CCA is the abundant desmoplastic stroma infiltration within the tumor, in which the presence of CAFs is responsible for the dense stroma of CCA [126]. By using immunohistochemical staining for α-SMA, the degree of CAFs infiltration in different CCA samples can be determined [127]. Other markers, such as FSP-1, PDGFR, can also be used to identify CAFs in CCA, among which positive FSP-1 has the highest expression rate in CAFs, reaching 84.5% [128]. Interestingly, however, unlike other solid tumors, such as breast and pancreatic cancer, where abundant stroma is associated with poor patient outcomes [129, 130], in ICC, patients with high proportion of stroma area exhibit a better disease-free survival, which indicates that desmoplastic stroma seems to exert a protective effect [131].

### The cellular origin of CAFs in CCA

The origin of CAFs in CCA is still not very clear, and previous studies suggested that it may be derived from activated HSCs or PFs [132, 133]. Recently, a study used Lrat-Cre-driven lox-stop-lox-TdTomato (TdTom) system to label HSCs in two ICC murine models, they found that in the context of ICC, 85%–95% of Col1a1-GFP + CAFs and 85%–93% of α-SMA + CAFs came from HSCs. Subsequently, they confirmed that HSCs-originated CAFs represented the subclusters with the most ligand-receptor interactions with tumor cells via scRNA-seq analysis of murine as well as human CCA samples [46].

Hence, given the unique properties of CCA, comprehending the activation status of CAFs in the stroma, specific CAFs subtypes, and the interaction of CAFs with surrounding cells provides new insights into the malignant progression and treatment of CCA.

## The roles of CAFs in CCA progression

Previous studies have shown that CAFs could produce various factors in the TME to promote the progression of CCA. For instance, SDF-1 secreted by CAFs binds to CXCR4 on the surface of CCA cells and mediates the invasion of CCA through the ERK1/2 and AKT pathways [134]. HGF could also be released by CAFs to stimulate CCA cells invasion in vitro assay [135]. In addition, some ECM components secreted by CAFs, such as periostin, tenascin-C, were involved in tumor migration and invasion [136, 137], and a variety of matrix metalloproteases (MMPs), including MMP1, MMP2, and MMP9, could also be produced by CAFs, which influenced tumor development via mediating ECM remodeling [138–140]. It is worth noting that CAFs in CCA are also heterogeneous, and different subtypes of CAFs can affect tumor development through distinct mechanisms. According to a recent study based on scRNA-seq analysis, CAFs in ICC can be categorized into three subpopulations, inflammatory and growth factor-enriched CAFs (iCAFs), myofibroblastic CAFs (myCAFs), and mesothelial CAFs (mesCAFs). iCAFs mediated ICC growth through the HGF-MET axis, while myCAFs promoted ICC progression by producing hyaluronan synthase 2 rather than type I collagen [46]. Another scRNA-seq analysis employed a negative selection strategy to enrich fibroblasts, dividing ICC-infiltrating CAFs into 5 subclusters, apart from myCAF and iCAF, vascular CAFs (vCAFs), antigen-presenting CAFs (apCAFs), EMT-like CAFs (eCAFs), and lipid metabolism-related fibroblasts are also classified [47], the molecular characteristics of each subgroup were summarized in Table 1.

### CAFs enhance chemoresistance of CCA cells

As mentioned above, CD90-positive CAFs secreted PIGF to promote angiogenesis in HCC. Likewise, PIGF is mainly expressed in stromal cells and is associated with poor prognosis in CCA. In vivo studies have shown that by blocking PIGF production in CAFs, the stiffness of the tumor could be weakened, thereby improving the hypoxic status, and enhancing the blood supply of the tumor, which was more conducive to the application of chemotherapeutic drugs [141]. The results suggested that by antagonizing some cytokines secreted by CAFs, its chemoresistance effect could be attenuated. Tyrosine kinase inhibitors (TKI), such as erlotinib, are administrated to treat CCA, however, they did not provide significant improvement of survival in clinical trials in CCA [142]. A recent study found that erlotinib-resistant CCA cells highly expressed insulin receptor (IR) and insulin-like growth factor (IGF) 1 receptor (IGF1R), and In vivo tumor formation model constructed from these resistant cells showed rich CAFs infiltration. Meanwhile, CAFs-secreted IGF2 stimulated IR/IGF1R signaling activation in resistant cells, which in turn promoted CAFs proliferation and activation for CCA-CAF interaction [143]. Additionally, in a 3D co-culture model, CAFs have a significant impact on reducing the sensitivity of CCA cells to chemotherapeutic drugs, including gemcitabine, cisplatin, and 5-fluorouracil (5-FU), but the insufficient effect on erlotinib [144]. The previous study has demonstrated that IL-6 secreted by CAFs could inhibit autophagy in CCA cells to stimulate CCA progression [145]. Moreover, IL-6 released by CAFs could educate neighboring CCA cells, rendering them less sensitive to chemotherapeutics via inhibiting the autophagy stress-response to the drug [146]. MiR-206 acted as a suppressor factor in liver cancer and played a suppressive role in the activation of HSCs, the crosstalk of CAFs with CCA cells reduced MiR-206 expression, which induced the conversion of NFs into CAFs and enhanced their secretion of IL6. When overexpressed MiR-206, the mutual interplay between CAFs and CCA was attenuated, and the resistance to gemcitabine was also been blockage [147].

### The role of CAFs on stemness in CCA

In most solid tumors, CSCs occupy only a small proportion, however, in CCA, CSCs account for up to 30% of the tumor bulk, indicating that CCA is a CSCs-based tumor [148]. Several CSCs surface markers have been identified in CCA, such as CD133, epithelial cell adhesion molecule (EpCAM), CD44, CD13, and CD90 [149–153]. The supportive ‘CSCs niche’ formed by the interaction of the abundant seeds ‘tumor cells and fertile soil ‘CAFs’ can maintain the proliferation and self-renewal of CSCs. According to the scRNA-seq data analysis mentioned above, one of the CAFs subsets, CD146-positive vascular CAFs (vCAFs), could secrete IL6 and significantly enhance the stemness ability of CCA [47]

In addition to direct regulation of CSCs, CAFs can also indirectly sustain tumor stemness by influencing the immune microenvironment. A previous study has shown that a subset of CAFs, FAP + CAFs, could recruit MDSCs into the TME by secreting CCL2 to exert immunosuppressive function [154]. Moreover, accumulating evidence has demonstrated that MDSCs could promote cancer stemness in a paracrine fashion [155]. Recently, a study showed that CAFs may indirectly regulate tumor stemness by educating MDSCs in the TME. By using an orthotopic ICC model, co-injection of CAFs and ICC cells in the livers of nude mice significantly induced the stemness of cancer, which could be attenuated by depletion of CAFs or MDSCs. Mechanistically, IL-6 and IL-33 secreted by CAFs stimulated the hyperactivated 5-lipoxygenase (5-LO) metabolism in CD33 + MDSCs, contributing to a large accumulation of downstream metabolite leukotriene B4 (LTB4). Abundant LTB4 acted on its receptor Leukotriene B4 receptor type 2 (BLT2) in ICC cells to promote ICC stemness via activation of PI3K/Akt-mTORC1 signaling [90]. This study also revealed that in addition to cytokines secreted by multiple cells that can enhance tumor stemness, amino acid metabolism is also involved in the regulation of tumor stemness.

### CAFs modulate immune responses in CCA

TILs in CCA also own their specific infiltration characteristics. According to the latest scRNA-seq data analysis of CCA, T cells and NK cells in the CCA were divided into 8 distinct subsets, and it was found that CD8 + T cells were in an exhausted status, and CD4 + Foxp3 + Treg cells were enriched in the TME while exhibiting a highly immunosuppressive feature [47]. CAFs also modulate the function of immune cells to drive an immunosuppressive microenvironment. Several studies have identified the interaction between CAFs and MDSCs. As mentioned above, FAP + CAFs could enhance MDSCs recruitment by secreting CCL2, and this tumor-promoting effect relied on MDSCs but independently of their immunosuppressive function [89]. After recruitment of MDSCs into the TME, CAFs set out to further educate MDSCs to enhance their stemness capacity via 5-LO/LTB4-BLT2 signaling [90]. Caveolin-1 (CAV1) has previously been reported to play a major role in cellular senescence, but its effect on tumors depends on the cancer type [156–158]. In ICC, CAV1 was highly expressed in CAFs, and CAV1 + CAFs were associated with poor prognosis in ICC patients. Immunohistochemical analysis showed that CAV1 + CAFs also correlated with infiltration of Foxp3 + TILs, which suggested that CAV1 + CAFs might recruit Tregs into the TME to mediate the prognosis of ICC [93]. Co-culturing of CAFs and CCA cells up-regulated CXCL5 expression in CCA cells, and secreted CXCL5 could promote the migration and invasion of CCA. Furthermore, CXCL5 had a direct recruitment effect on TANs in HCC [159]. Similarly, CD66 + TANs were positively correlated with CXCL5 in CCA tissues, and CXCL5 secreted by stromal cells mediated TANs chemotaxis through activation of PI3K-AKT and ERK1/2 pathways [91, 92].

### CAFs regulate angiogenesis in CCA

Unlike HCC, CCA is a hypovascular tumor, and the dense stroma constructed by abundant CAFs induces the collapse of tumor blood vessels to form a hypoxic microenvironment. In addition to structural factors, CAFs can also secrete cytokines to mediate the angiogenesis of CCA. The PDGF family has been reported to promote the HSCs migration and proliferation, among which PDGF-D secreted by CCA cells promoted CAFs recruitment via PDGFRβ and Rho GTPase and JNK activation in CCA [160]. Hypoxia-induced PDGF secretion from CCA cells could bind to the receptors PDGFRβ expressed on the CAFs, thereby stimulating CAFs to produce VEGF-A and VEGF-C. In CCA, CAFs and lymphatic endothelial cells were spatially adjacent to each other, moreover, the lymphatic vessels provided an important dissemination pathway for the metastasis of CCA cells. Ultimately, VEGF secreted by CAFs promoted the chemotaxis and assembly of lymphatic endothelial cells to form the appropriate lymphatic vasculature that supported CCA invasion [161]. PlGF, a member of the VEGF family, which has been documented to enhance VEGF-driven angiogenesis by binding to VEGFR1 and Nrp1 on endothelial cells [162], was recently demonstrated to be produced by CAFs to deteriorate the hypoxic state in CCA through enhancing the stiffness of the stroma, compressing the tumor vessels [141]. In addition, it was worth noting that, unlike VEGF, PIGF was only up-regulated in pathological conditions such as hypoxia, and undetectable in healthy conditions [163]. Therefore, PIGF could be targeted to selectively inhibit the angiogenesis in pathological conditions without interfering normal blood vessel growth.

### Activation of CAFs in CCA

Similarly, the mutual communications between CCA cells and CAFs also make CCA cells secrete some cytokines to promote the activation and function enhancement of CAFs to further aggravate tumor progression. This vicious cycle could be initiated by CAFs-generated heparin-binding epidermal growth factor (HB-EGF), which bound to epidermal growth factor receptor (EGFR) on CCA cells to promote CCA cells invasion through (ERK)1/2 and STAT3 signaling pathways. Activation of EGFR in CCA cells could produce TGF-β, which facilitated CAFs activation and HB-EGF production [164]. Previous studies reported that the interaction of tumor necrosis factor-like weak inducer of apoptosis (TWEAK) and its receptor fibroblast growth factor-inducible 14 (Fn14) could lead to the progression of liver fibrosis by modulating myofibroblast proliferation [165]. In CCA, TWEAK overexpression promoted the proliferation of collagen-producing CAFs, which expressed Fn14 in a substantial proportion of CCA patients. Accordingly, the TWEAK/Fn14 signaling axis may serve as an early driver of tumor niche development to promote tumor growth in CCA [166]. CCA-derived exosomes also mediate CAFs activation, the expression level of miR-34c was decreased in exosomes derived from CCA, and downregulation of miR-34c could induce CAFs activation by targeting the Wnt signaling pathway in CCA [167]. Zinc Finger E-Box Binding Homeobox 1 (ZEB1) as a transcription factor was associated with poor prognosis in ICC and ZEB1 expression in ICC cells induced tumor EMT and stemness phenotype. Besides, ZEB1 was involved in the interplay between CCA cells and CAFs by regulating the expression of HGF and IL6 to promote CCA progression. In vitro studies showed that CTGF in the supernatants of ZEB1-overexpressing CCA cells promoted the proliferation of myofibroblasts. Mechanistically, ZEB1 upregulated CTGF expression by directly binding to the promoter of CTGF. Furthermore, ZEB1 was also expressed on CAFs and contributed to the activation of stromal CAFs [168]. PIGF secreted by ICC cells promoted the activation of CAFs to acquire a myofibroblast-like phenotype via AKT/NF-kB pathway, then activated CAFs could, in turn, promote ICC cells invasion [141]. Programmed cell death ligand 1 (PD-L1) as a known immune checkpoint molecule could induce immune tolerance in the TME, however, a recent study found that PDL1 expressed by HSCs was required for the transformation of HSCs into myofibroblasts but was independent of the immunosuppressive function of PDL1. Mechanistically, the PD-L1 extracellular domain bound to TGF-β receptors II (TβRII) of HSCs to protect TβRII from lysosomal degradation, and the cytoplasmic domain of PD-L1 protected TGF-β receptors I (TβRI) mRNA from degradation by the RNA exosome complex in HSCs [169].

## Conclusions

**Fig. 2:**
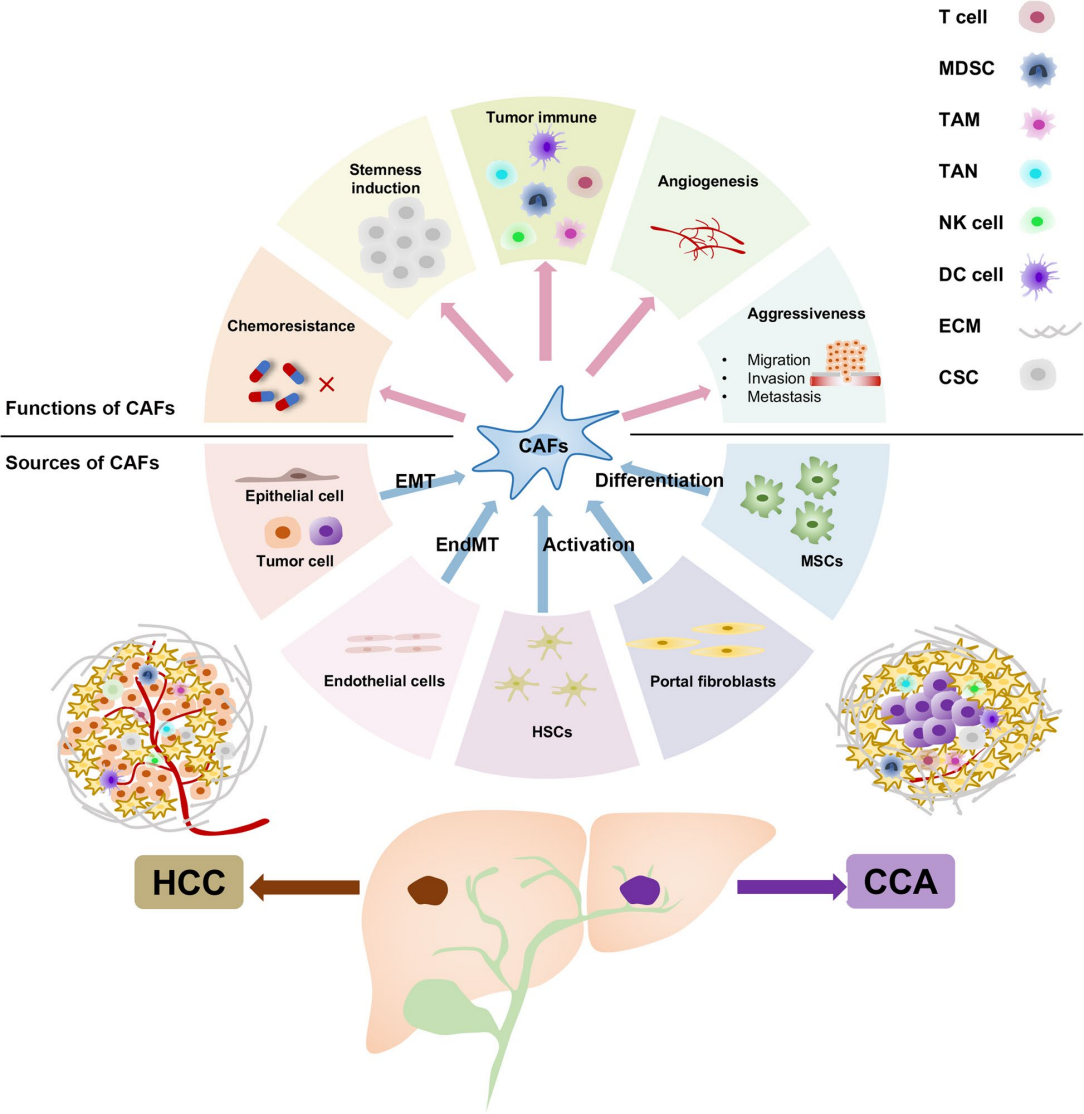
The origin and the role of CAFs in liver cancer. Schematic illustration of potential cellular origins of CAFs, including epithelial cells, endothelial cells, tumor cells, HSCs, portal fibroblasts, and MSCs. The upper part of the picture shows the the influence exerted by CAFs in the TME, including chemoresistance, stemness induction, tumor immune, angiogenesis, and aggressiveness

In recent years, accumulating studies have emphasized the non-negligible role of CAFs as the main component of tumor stroma in the TME of liver cancer. In this review, we mainly summarized the cellular origin of CAFs and several aspects that CAFs implicated in the progression of liver cancer: chemotherapy resistance, tumor stemness maintenance, induction of immunosuppressive microenvironment, tumor angiogenesis, and activation of CAFs by liver cancer cells (Fig. 2), which were summarized in Table 3. Using modern scRNA-seq analysis and gene lineage tracing techniques have identified HSCs as the major cellular source of CAFs and also revealed the heterogeneity of CAFs within liver cancer. However, it should be noted that the use of scRNA-seq analysis to identify specific subsets of CAFs is based on the transcriptional expression levels of signature genes, and this analysis does not totally reflect the protein expression profiling. Therefore, it is the necessity to explore the distinct function of CAFs subpopulations, including the use of 3D cultures, organoids that more closely mimic the in vivo microenvironment, and transgenic mouse models and patient-derived xenografts models.

**Table 3:** The effects and mechanisms of CAFs on HCC cells/CCA cells

	**HCC-CAFs**		**CCA-CAFs**	
	**Mediator**	**Effects and mechanisms in HCC**	**Mediator**	**Effects and mechanisms in CCA**
**chemoresistance**	HGF	CAF-derived HGF enhances the resistance of HCC cells to sorafenib and cisplatin by upregulating the expression of CD73 [18]	PIGF	Antagonizing CAF-secreted PIGF can alleviate chemoresistance effects [141]
	CAFs-supernatants	CAF supernatant induces RCN1 expression in HCC cells, thereby enhancing resistance to sorafenib via the IRE1α-XBP1s pathway [48]	IGF2	CAFs-secreted IGF2 stimulated IR/IGF1R signaling activation in erlotinib-resistant CCA cells [143]
			IL6	CAF-secreted IL6 attenuates the sensitivity of CCA cells to chemotherapeutic drugs by inhibiting autophagy in CCA cells [146]
**Cancer stemness**	IL6	CAFs-secreted HGF and IL6 enhanced stemness of CD24 + HCC cells through activated STAT3 pathway [57]	IL6	CD146-positive vascular CAFs (vCAFs)-secreted IL6 enhanced the stemness of CCA [47]
	HGF	CAFs-derived HGF enhanced stemness via (ERK)1/2–FRA1–HEY1 pathway [51]	MDSCs	CAFs indirectly regulated tumor stemness by recruiting MDSCs in the TME of CCA [90]
		HGF induces KRT19 expression in HCC cells via the MET-ERK1/2-AP1 and SP1 axis, thereby promoting stemness maintenance [54]		
	FSTL1	CAFs-derived FSTL1 promoted the stemness through the AKT/mTOR/4EBP1 signaling pathways [60]		
	CLCF1	CAF-secreted CLCF1 enhances stemness by promoting the secretion of CXCL6 and TGF in HCC cells [61]		
	FOXQ1	CAF promotes HCC initiation via the FOXQ1/NDRG1 axis [63]		
	RvD1	RvD1 inhibits CAF-secreted COMP to antagonize the stemness effect via FPR2/ROS/FOXM1 signaling [69]		
**angiogenesis**	VEGF	CAFs-derived VEGF promote the angiogenesis via EZH2 /VASH1 pathway [94]	VEGF	VEGF secreted by CAFs promoted the chemotaxis and assembly of lymphatic endothelial cells [161]
	PIGF	CD90 positive CAFs have a strong correlation with PIGF expression in HCC tissues [96]	PIGF	PIGF produced by CAFs could compress the tumor vessel and deteriorate the hypoxic state in CCA [141]
	TGF-β and SDF1	CAFs-derived TGF-β and SDF1 facilitated VM formation [97]		
	prolargin	CAFs-secreted prolargin inhibited the angiogenesis of HCC [45]		
	SPARCL1	SPARCL1-positive fibroblasts could maintain the self-stabilization of blood vessels [41]		

Based on the biological research of CAFs in liver cancer, currently, many novel therapeutic strategies targeting CAFs have been explored and developed, such as targeting the precursors of CAFs, mainly by inhibiting the activation of HSCs. Alternatively, blocking the tumor-promoting factors that CAFs-produced and the CAFs-mediated signaling pathways, such as developing inhibitors against IL6, CTGF, TGF-β, and SMAD, p-STAT3 signaling, etc. In addition, the application of nano-delivery systems makes it possible to precisely target and eliminate tumor-promoting CAFs and tumor cells. However, due to the lack of tumor clinical trials and considering the existence of tumor-suppressive CAFs, more efforts should be devoted to ensuring the clinical safety and translation of various nanoparticle formulations.

In conclusion, based on the in-depth research on the diverse functions of CAFs, the more precise elimination of tumor-promoting CAFs subsets or systemic combination therapy could become an effective strategy for the liver cancer treatment.

## Data Availability

Not applicable.

## Abbreviations

HCC: Hepatocellular carcinoma
CCA: Cholangiocarcinoma
TME: Tumor microenvironment
CAFs: Cancer-associated fibroblasts
HBV: Hepatitis B virus
HCV: Hepatitis C virus
NAFLD: Nonalcoholic fatty liver disease
NASH: Nonalcoholic steatohepatitis
ECM: Extracellular matrix
PDAC: Pancreatic ductal adenocarcinoma
MMP: Matrix metalloproteinase
α-SMA: α-Smooth muscle actin
FAP: Fibroblast activation protein
PDGF: Platelet-derived growth factor
FSP-1: Fibroblast-specific protein 1
TGF-β: Transforming growth factor-β
HGF: Hepatocyte growth factor
IGF: Insulin-like growth factor
IL-6: Interleukin-6
HSCs: Hepatic stellate cells
MSCs: Mesenchymal stem cells
scRNA-seq: Single-cell RNA sequencing
Lrat-Cre: Lecithin retinol acyltransferase-cyclization recombination enzyme
Tcf21: Transcription factor 21
EMT: Epithelial-mesenchymal transition
EndMT: Endothelial to mesenchymal transition
PFs: Portal fibroblasts
LPA: Lysophostatidic acid
RCN1: Reticulocalbin 1
BAFF: B-cell activating factor
CSCs: Cancer stem cells
FSTL1: Follistatin-like 1
CLCF1: Cardiotrophin-like cytokine factor 1
ERK: Extracellular signal-regulated kinase
LXRs: Liver X receptors
RvD1: Resolvin D1
TILs: Tumor-infiltrating lymphocytes
TAMs: Tumor-associated macrophages
TANs: Tumor-associated neutrophils
Tregs: Regulatory T cells
MDSCs: Myeloid-derived suppressor cells
TIME: Tumor immune microenvironment
LPS: Lipopolysaccharides
IFN-γ: Interferon-γ
TNF: Tumor necrosis factor
CXCL12: C‑X‑C motif chemokine ligand 12
PAI‑1: Plasminogen activator inhibitor‑1
GAS6: Growth arrest-specific protein 6
PGE2: Prostaglandin E2
IDO: Indoleamine 2,3-dioxygenase
ROS: Reactive oxygen species
SDF: Stromal cell-derived factor
MCP-1: Monocyte chemotactic protein-1
COX-2: Cyclooxygenase-2
VEGF: Vascular endothelial growth factor
HUVECs: Human umbilical vein endothelial cells
PlGF: Placental growth factor
VM: Vasculogenic Mimicry
CTGF: Connective tissue growth factor
TIMPs: Matrix metalloproteinases
SULF2: Sulfatase 2
ER: Endoplasmic reticulum
IREα: Inositol requiring enzyme 1α
SASP: Senescence associated secretory phenotype
BMP4: Bone morphogenetic protein 4
EVs: Extracellular vesicles
ICC: Intrahepatic CCA
PCC: Perihilar CCA
DCC: Distal CCA
TKI: Tyrosine kinase inhibitors
IGF2: Insulin-like growth factor 2
EGFR: Epidermal growth factor receptor
ZEB1: Zinc Finger E-Box Binding Homeobox 1
PD-L1: Programmed cell death ligand 1
